# Supplementary material for: The Identification of Viral Pathogens in a Physostegia virginiana Plant Using High-Throughput RNA Sequencing
Source: Viruses. 2023 Sep 21;15(9):1972. doi: 10.3390/v15091972 (PMC10534606; doi:10.3390/v15091972)
Supplement: Supplementary file 1 [file viruses-15-01972-s001.zip › Supplementary Materials -tables.pdf]

Supplemental tables

Table S1. Detection primers used in this study.

Virus name	Primer name	Gene sequence	Amplified gene length(bp)
AMV	AMV-F	CCGCTTGGTATGGTTATCTTATC	468
	AMV-R	ATTATCCACACGCAAGGGCACTCAA	
PVCaV1	RNA1-FT	AGGAGATTTCCATGTCAAGTAGGTGT	468
	RNA1-RT	ATTAAACTGGCCTGCAATCGTAGC	
	RNA2-FT	TCCCTCTAAACATCACGCCTGG	518
	RNA2-RT	GTACGTGACAGTGCCAATTCGTT	
PVCV1	PVCV1-F	GCTTAACTCAGGATTAGTACAATCTAT	517
	PVCV1-R	GTGATGACTATTGCTTAAGGCAT	
PVCV2	PVCV2-F	CAAGCAGATTACAGATTTGGTTGTTACG	2195
	PVCV2-R	TATCGTTGCTGTGGTAGTTTAACTGC	
Contig-PVF	20685-F	TGGATGACAGCCACTTTCTTAGGATC	510
V- 20685	20685-R	TCAATCCAAAACCTTCCAAATCAAAAA	

Table S2. Specific primers used to amplify DNA/RNA sequences of viral contigs in this study.

Virus name	Primer name	Gene sequence	Amplified gene length(bp)
(***Fabavirus***)			
PVCaV1	RNA1-1F	GTGGTGTGATTCAAAGCGCACAA	1529
	RNA1-1R	AATCGCGGCAGTTTGTAGTTCTTT	
	RNA1-2F	GTCTAACCACCGGGGTTCAGTG	1450
	RNA1-2R	CCGAATGTTACGGAATACATAATCAG	
	RNA1-3F	CTCTGTGGAATTTGGGAACTGGAC	1481
	RNA1-3R	CAGAAACTTCCTCCTAACAACCAAGT	
	RNA1-4F	CCCAAGACACGATTATTTCTGTGC	1535

	RNA1-4R	ATACACACTGGACTTTAATCAGTCCTT	
	RNA2-1F	CATCGTGCAGCGTTCTGGAAT	1457
	RNA2-1R	TGTTTAGAGGGATGTTTGCACGAACA	
	RNA2-2F	CAGGGTCAAGCTTTAATGGAAGAGGAT	1783
	RNA2-2R	CAACAGACGTGACCTTGTCAAGCT	
(***Caulimovirus***)			
PVCV1	1-1-F	TGGTATCAGAGCCATGGAAAGAG	4000
	1-1-R	ACAGTTGTTGGATCTATGAGCTTAATG	
	1-2-F	GTTTAGAGAGGGGGAGATTGGAGTT	3949
	1-2-R	GGTTTTTAGAAACGCCTGAGGCTT	
PVCV2	2-1-F	TGGTATCAGAGCATGTTCGCTGATC	4300
	2-1-R	TATTAGGGAGGTTGTGACCATCACC	
	2-2-F	AGAAATACTTGAACTGGACAAGGTTT	4276
	2-2-R	AAGTGGGGCCAAGCTTACG	
(***Fijivirus***)			
Contig-PVFV-20685	20685-F1	CTAAAAAGCATGTAAGATGGATGAC	983
	20685-R1	ATCCTGAGATTGGTTTCGTACCATG	
	20685-F2	AGGATCAGTTTCAGTTCTTGAAGAGGG	1514
	20685-R2	CCGAAACATCCATTGATCTCCCG	
	20685-F3	AGCGGTTCATGCAGCTAAAGTT	2081
	20685-R3	CTACGGAACATTATAAATACCTAAATTA	
Contig-PVFV-5043	5043-F1	GAAGACCAAACCACTCAACCAGT	934
	5043-R1	CTTCAGTTACTTGACGTCCTACCCT	
	5043-F2	CTGAGGATGAAGCTTTTAGGGTAGG	1331
	5043-R2	TGCCTGACCCAATAAATTCCGTTG	
	5043-F3	CCACAAAATCAACGGAATTTATTGGG	1480
	5043-R3	GTTCCTGAAACCTACTCGCGC	
Contig-PVFV-3081	3081-F1	GTTAAACTTAGGAAGTTTCCAATTATAC	1290
	3081-R1	TTTGGAACGATATCATATGTCTCGAC	

	3081-F2	ATATTTGCGTGCTAGGTTTAACAAT	1305
	3081-R2	ATATCCCTATGACCAAATTTAAACATC	
	3081-F3	AACATCTTGCAGTCAATTCAACA	1321
	3081-R3	TATAGGTTGTTCCCCTTAAAGTCCTG	
Contig-PVFV-8459	8459-F1	CCATCTTCGATTTGATCCGGC	2028
	8459-R1	GGTCCCAATTTTCACTCTGCCT	
	8459-F2	GGTCAAATTGGTGATTCAGATG	2099
	8459-R2	GTCATATCATCCTGAGAAGGGTT	
Contig-PVFV-36481	36481-F1	CTCTCATTAAAATTAACTGTGGGTAAG	1212
	36481-R1	ACTCAACAGAACAGAATTTTGTTG	
	36481-F2	GTTGTTCGATCAAGTTATGCTAAGA	1856
	36481-R2	GTGACCGGTCATGTCCTCGT	
Contig-PVFV-2375	2375-F1	CGTGGACGAAAAGAACCGAT	1992
	2375-R1	CCGTGAACATATCTCCATACCATGAA	
Contig-PVFV-7856	7856-F1	TCTGACATGTCTAGCTCGAATACAG	1805
	7856-R1	CGGAATACAGAATTAAACTGCACAGG	
	7856-F2	GTGTGGACTTGATTCTGGATTTACC	1215
	7856-R2	TTTAAATCTTATTTTGGCGGGGCCTT	
Contig-PVFV-33929	33929-F	CCTCCTTGAGAACTCATAGCGGA	1631
	33929-R	CCCACCACCTCGCTTACAAAG	
Contig-PVFV-25766	25766-F	CTACGATGGATCTTACAACAGTTCAAG	2028
	25766-R	GTGCTTATCAACACGGGATTCGAAT	

Table S3. Specific primers used to amplify the full-length genomes of the three novel viruses

Virus name	Primer name	Gene sequence
PVCaV1	RNA1-5'GSP-681	ATGCAAAAGTGTTGGTGCACGCACCATG
	RNA1-3'GSP-5324	GCAATTGATGGCGAACGTGTACGCTGCG
	RNA2-5'GSP-559	CCGTGGTCTCTGGGAGAATACTGTG

	RNA2-3'GSP-2796	ACACTCTCCCGTGACGCGGATT
PVCV1	1-F1	CAGGAAGAAGGCAATAATCTAG
	1-R1	AAGTGCATCTTCTCTTTTGTTG
	1-F2	GGGTAAGCGGTAAACATAAAT
	1-R2	AAATTTGGACTGTCAGAGCCA
PVCV2	2-F1	AAGGGCGTATCTTGTACTGTTC
	2-R1	ACAAACACATAGCAGAGAGAAG
	2-F2	TGCGAACAAGTAAGTTCTTGG
	2-R2	AGAACCATCCAACTACAAGGA

Table S4. Viruses used to analyze the amino acid identity and construct phylogenetic trees of PVCaV1

Virus name	Abbreviation	Family	Accession No.
Broad bean wilt virus 1	BBWV1	*Fabavirus*	AB084450 & AB084451
Broad bean wilt virus 2	BBWV2	*Fabavirus*	AF225953 & AF225954
Cucurbit mild mosaic virus	CuMMV	*Fabavirus*	EU881936 & EU881937
Peach leaf pitting-associated virus	PLPaV	*Fabavirus*	MK460243 & MK460244
Gentian mosaic virus	GeMV	*Fabavirus*	AB084452 & AB084453
Grapevine fabavirus	GFabV	*Fabavirus*	KX241482 & KX241485
Lamium mild mosaic virus	LMMV	*Fabavirus*	KC595304 & KC595305
Prunus virus F	PrVF	*Fabavirus*	KX192389 & KX192393
Cowpea mosaic virus	CPMV	*Comovirus*	X00206 & X00729
Bean pod mottle virus	BPMV	*Comovirus*	U70866 & M62738
Cherry rasp leaf virus	CRLV	*Cheravirus*	AJ621357 & AJ621358
Strawberry mottle virus	SMoV	*Sadwavirus*	AJ311875 & AJ311876
Lettuce star mosaic virus	LSMV	*Sequivirus*	MT348706

Beet ringspot virus	BRSV	*Nepovirus*	D00322 & X04062
Cherry leaf roll virus	CLRV	*Nepovirus*	FR851461 & FR851462
Strawberry latent ringspot virus	SLRSV	*Stralarivirus*	AY860978 & AY860979
Motherwort yellow mottle virus	MYMoV	*Torradovirus*	KM229700 & KM229701
Oat mosaic virus	OMV	*Bymovirus*	NC_004016.1

Table S5. Viruses used to analyze the nucleotide and amino acid identity and construct phylogenetic trees of PVCV1 and PVCV2

Virus Name	Abbreviation	Family	Accession No.
Angelica bushy stunt virus	AnBSV	*Caulimovirus*	KU508800
Atractylodes mild mottle virus	AMMV	*Caulimovirus*	ALD49090
Carnation etched ring virus	CERV	*Caulimovirus*	X04658
Cauliflower mosaic virus	CaMV	*Caulimovirus*	V00141
Dahlia mosaic virus	DaMV	*Caulimovirus*	JX272320
Figwort mosaic virus	FMV	*Caulimovirus*	X06166
Lamuim leaf distortion-associated virus	LLDAV	*Caulimovirus*	EU554423
Horseradish latent virus	HLV	*Caulimovirus*	JX429923
Mirabilis mosaic virus	MMV	*Caulimovirus*	AF454635
Soybean putnam virus	SPuV	*Caulimovirus*	JQ926983
Strawberry vein banding virus	SVBV	*Caulimovirus*	X97304
Banana streak GF virus	BSGFV	*Badnavirus*	AY493509
Badnavirus castaneae	ChMV	*Badnavirus*	MT269853
Commelina yellow mottle virus	CoYMV	*Badnavirus*	X52938
Epiphyllum virus 4	EpV4	*Cavemovirus*	MN153807
Sweet potato collusive virus	SPCV	*Cavemovirus*	HQ694978
Cassava vein mosaic virus	CsVMV	*Cavemovirus*	U59751
Dioscorea nummularia-associated virus	DNUaV	*Dioscovirus*	MG944237
Petunia vein clearing virus	PVCV	*Petuvirus*	U95208
Rose yellow vein virus	RYVV	*Rosadnavirus*	JX028536
Tobacco vein clearing virus	TVCV	*Solendovirus*	AF190123
Sweet potato vein clearing virus	SPVCV	*Solendovirus*	HQ694979

Soybean chlorotic mottle virus	SbCMV	*Soymovirus*	X15828
Rice tungro bacilliform virus	RTBV	*Tungrovirus*	X57924
Blueberry fruit drop-associated virus	BFDaV	*Vaccinivirus*	KT148886

Table S6. Viruses used to analyze the nucleotide and amino acid identity and construct the phylogenetic tree of PVFV

Virus name	Family/Genus	Accession No.
Mammalian orthoreovirus 1	*Spinareoviridae/Orthoreovirus*	KX263307
Mammalian orthoreovirus 3	*Spinareoviridae/Orthoreovirus*	HM159613
Ndelle virus	*Spinareoviridae/Orthoreovirus*	AF368033
Avian orthoreovirus	*Spinareoviridae/Orthoreovirus*	AB914761
Mahlapitsi Orthoreovirus	*Spinareoviridae/Orthoreovirus*	KU198604
Reptilian orthoreovirus	*Spinareoviridae/Orthoreovirus*	KT696549
Broome reovirus	*Spinareoviridae/Orthoreovirus*	GQ258979
Chum salmon reovirus/aquareovirus A	*Spinareoviridae/Aquareovirus*	AF418295
Golden shiner reovirus/aquareovirus C	*Spinareoviridae/Aquareovirus*	AF403399
Cryphonectria parasitica mycoreovirus 1	*Spinareoviridae/Mycoreovirus*	AY277888
Rosellinia necatrix mycoreovirus 3	*Spinareoviridae/Mycoreovirus*	AB102674
Eyach virus	*Spinareoviridae/Coltivirus*	AF282467
Colorado tick fever virus	*Spinareoviridae/Coltivirus*	AF133428
Rice black-streaked dwarf virus	*Spinareoviridae/Fijivirus*	AJ294757
Maize rough dwarf virus	*Spinareoviridae/Fijivirus*	KU984966
Southern rice black-streaked dwarf virus	*Spinareoviridae/Fijivirus*	FN563989
Mal de Rio Cuarto virus	*Spinareoviridae/Fijivirus*	AF499925
Fiji disease virus	*Spinareoviridae/Fijivirus*	AY029520
Nilaparvata lugens reovirus	*Spinareoviridae/Fijivirus*	D49693
Aedes pseudoscutellaris reovirus	*Spinareoviridae/Dinovernavirus*	DQ087277
Rice ragged stunt virus	*Spinareoviridae/Oryzavirus*	U66714
Dendrolimus punctatus cypovirus 1	*Spinareoviridae/Cypovirus*	AY147187
Lymantria dispar cypovirus 14	*Spinareoviridae/Cypovirus*	AF389452
Tobacco mosaic virus	*Virgaviridae/Tobamovirus*	V01408.1

Table S7. Sixteen contig obtained from RNA-seq

Contig name	Consensus length	Total read count	Average coverage
Contig- AMV-RNA1	3631	406930	16787.99
Contig-AMV-RNA2	2590	101655	5880.81
Contig-AMV-RNA3	2039	184602	13561.07
Contig-PVCaV1-RNA1	5801	230999	5942.10
Contig-PVCaV1-RNA2	3341	447315	19739.84
Contig-PVCV1	7765	104146	2005.81
Contig-PVCV2	7887	30954	587.30
Contig-PVFV-36481	2946	236	11.74
Contig-PVFV-8459	3430	158	6.44
Contig-PVFV-3081	3457	204	8.80
Contig-PVFV-5043	3738	231	9.23
Contig-PVFV-20685	4356	270	9.14
Contig-PVFV-25766	2015	241	17.72
Contig-PVFV-2375	1976	181	13.60
Contig-PVFV-7856	2936	201	10.19
Contig-PVFV-33929	1650	116	10.27
