# Supplementary material for: The Identification of Viral Pathogens in a Physostegia virginiana Plant Using High-Throughput RNA Sequencing
Source: Viruses. 2023 Sep 21;15(9):1972. doi: 10.3390/v15091972 (PMC10534606; doi:10.3390/v15091972)
Supplement: Supplementary file 1 [file viruses-15-01972-s001.zip › Supplementary Materials-Figure S1 and S2.pdf]

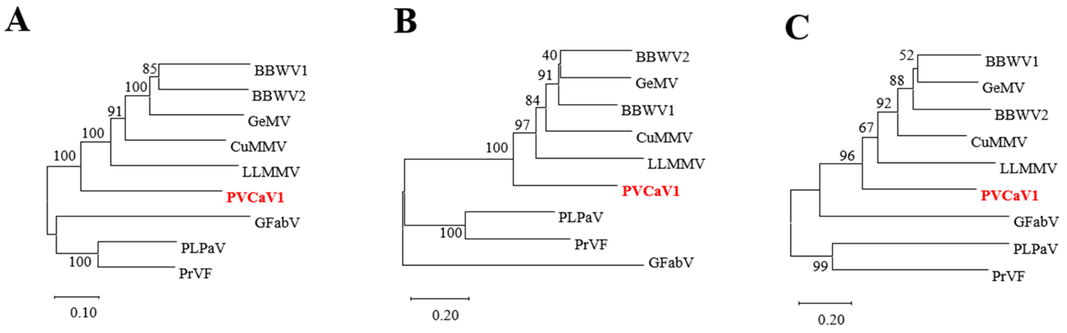

Figure S1. Phylogenetic trees of PVCaV1 and 8 representative viruses from the genus *Fabavirus* based on RdRp (A), LCP (B) and SCP (C). The phylogenetic tree was constructed using the Neighbor-Joining method with 1000 bootstraps. PVCaV1 is shown in red.

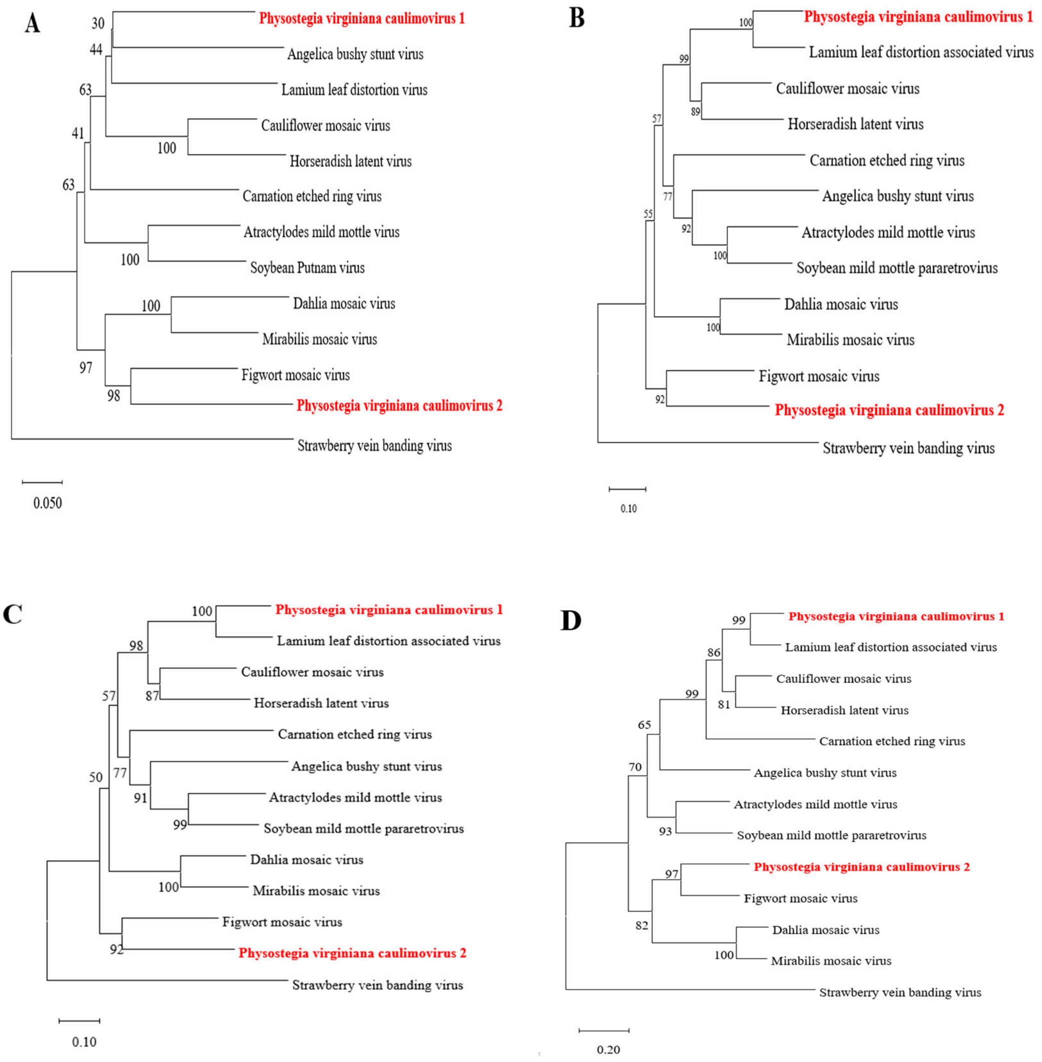

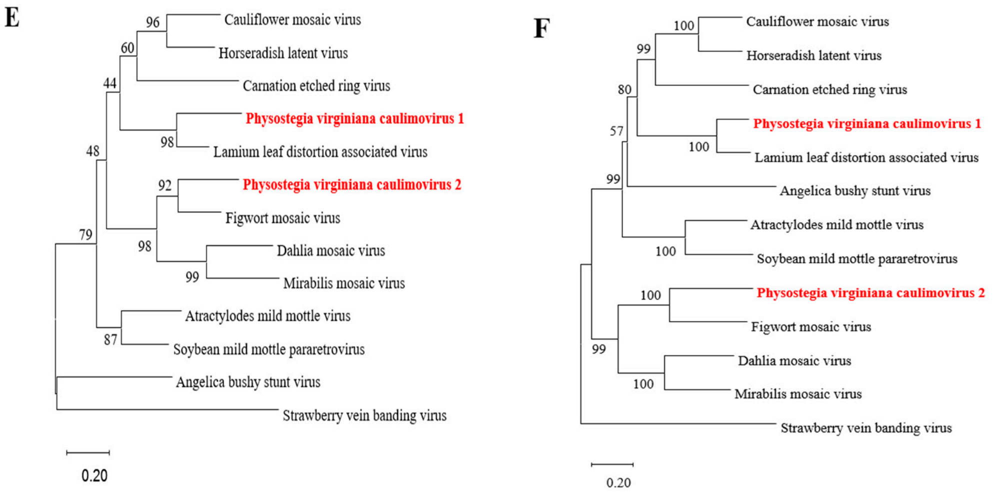

Figure S2. Phylogenetic trees based on PP (A), MP (B), CP (C), ATF (D), DNAb (E), and IB (F) amino acid sequences of PVCV1, PVCV2 and 11 other viruses from the genus *Caulimovirus*. The phylogenetic tree is constructed using the Neighbor-Joining method with 1000 bootstraps. PVCV1, PVCV2 is shown in red. The bootstrap values are indicated adjacent to the nodes. Accession numbers of these sequences are listed in Table S3.
